# Daily activity accumulation patterns and depressive symptoms among adolescents: a latent profile approach

**DOI:** 10.3389/fpsyg.2025.1683685

**Published:** 2025-10-15

**Authors:** Yuwei Liu, Nan Zheng, Huan Chen, Guo Liang, Ting Li, Yanping Qiu

**Affiliations:** School of Physical Education and Sport Training, Shanghai University of Sport, Shanghai, China

**Keywords:** adolescence, activity patterns, latent profile analysis, depressive symptoms, network analysis

## Abstract

**Objective:**

This study aims to identify and characterize daily activity accumulation patterns (bouts of physical activity and sedentary behavior) among adolescents and then to explore the associations between these groups and depressive symptoms.

**Methods:**

A total of 521 adolescents aged 13–18 years from Wuhan and Changsha, China, were included. Bouts of physical activity (PA) and sedentary behavior (SED) were measured using accelerometers. The Center for Epidemiologic Studies Depression Scale was used to assess participants’ depressive symptoms. Latent profile analysis was employed to identify distinct groups based on their activity patterns.

**Results:**

Three distinct groups were identified: “Prolonged sitters” (*n* = 149, 28.6%), “Sitters” (*n* = 224, 43.0%), and “Movers” (*n* = 148, 28.4%). After adjusted controlling for potential confounders, compared with the prolonged sitters, “Movers” [*β* (95% CI) = −3.6 (−5.912, −1.388)] exhibited the lowest score of depressive symptoms, followed by the “Sitters” [*β* (95% CI) = −2.3 (−4.240, −0.325)].

**Conclusion:**

The synergistic effect of strategies to reduce total SED duration by limiting SED bouts to 30 min or less and increasing light physical activity (LPA) may also be effective in alleviating depressive symptoms in adolescents.

## 1 Introduction

Depression is one of the most common mental disorders and has become a significant global public health concern. Adolescence is a critical period with a high prevalence of depression, and the Global Burden of Disease Study demonstrated that approximately 46 million adolescents worldwide suffer from depression, representing an increase of 21.67% compared to data from 1990 (Yang et al., 2024). In China, the results of the most representative study on mental health development indicated that the prevalence of depressive symptoms among adolescents increased from 7.4% in early adolescence to 25% in late adolescence (Fu and Zhang, 2021). Depression that appears in adolescence may develop into a chronic condition or recurrent episodes that continue into adulthood (Clayborne et al., 2019). Therefore, exploring modifiable factors associated with depressive symptoms in adolescents holds significant importance for prevention and intervention.

Individuals’ daily waking activities primarily comprise light physical activity (LPA), moderate-to-vigorous physical activity (MVPA), and sedentary behavior (SED). These behaviors have been recognized as significant and modifiable risk factors (Grgic et al., 2018; Rosenberger et al., 2019). Previous studies have predominantly examined the independent associations of physical activity (PA) and SED bouts with health outcomes, finding that increasing PA bouts or reducing SED bouts may lower health risks (Jakicic et al., 2019; Werneck et al., 2019). However, recent scholars have suggested that focusing solely on the independent health effects of bouts of PA or SED may be too narrow (Chinapaw et al., 2019; Verswijveren et al., 2020). This is because these two behaviors continuously alternate and interact during waking hours, forming unique activity patterns for everyone (Chinapaw et al., 2019; Cao et al., 2020). Importantly, these distinct activity patterns are closely related to mental health, regular PA can alleviate depressive symptoms, whereas elevated depressive symptoms may reduce PA participation (Lubans et al., 2016; Pearce et al., 2022). Therefore, it is necessary to categorize activity patterns with similar characteristics and conduct a comprehensive analysis of their features to explore their relationship with depressive symptoms in adolescents in greater depth.

One approach to achieving this goal is to adopt data-driven, person-centered methods such as latent profile analysis. Compared to variable-centered methods that solely examine overall relationships between variables, latent profile analysis offers a distinct advantage in identifying groups of individuals with similar characteristics (Collins and Lanza, 2010). This not only deepens our understanding of adolescents’ activity accumulation patterns but also enables the design of more targeted interventions based on group characteristics (Howard and Hoffman, 2018).

To the author’s knowledge, only one study has identified groups with similar activity accumulation patterns using latent profile analysis and examined the relationship between these patterns and adolescent health outcomes. Verswijveren et al. (2020) classified 1,219 Australian adolescents (7–13 years) into “prolonged sitters,” “breakers,” and “prolonged movers” based on PA and SED bouts characteristics. The “breaker” pattern was most common and associated with lower BMI and waist circumference. However, this study primarily focused on physical indicators without examining the role of activity patterns in mental health domains. It also failed to comprehensively incorporate the duration and frequency of PA and SED bouts, which reflect the cumulative characteristics of daily activity. Furthermore, the study sample was limited to Australian adolescents, and the applicability of its conclusions to other cultural and social contexts remains to be verified.

In China, adolescents show low PA and high SED, with only 14% meeting the WHO guideline of ≥60 min MVPA daily and over 93% exceeding 2 h of SED (Liu et al., 2023). The academically driven sedentary lifestyle highlights the need to investigate cumulative activity patterns in this context. And there has been no existing research examining adolescents’ activity patterns and their health effects. Accordingly, this study aimed (1) to identify adolescent activity patterns using latent profile analysis and (2) to examine their associations with depressive symptoms, independent of total SED, MVPA, and LPA duration and frequency. The findings are expected to provide new insights into how daily activity accumulation patterns influence adolescent mental health and offer evidence to inform targeted intervention strategies in the Chinese context.

## 2 Materials and methods

### 2.1 Participants

This study was conducted between March and June of 2023 and was approved by the Institutional Review Board of Shanghai University of Sport (102772023RT090). Three secondary schools (i.e., Grades 7–8 and 10–11 with students aged 13–18) were selected from Wuhan and Changsha cities in China. One or two classes were randomly selected from Grades 7–8 and 10–11 in each secondary school. Grade 9 and Grade 12 students were excluded from the study because of the promotion exam. A total of 20 classes with 848 students were invited to participate in the study, and after sending an informed consent form to their parents, all 734 students agreed to participate (86.55% response rate). The following students were excluded from the study: those with severe chronic illnesses (e.g., cardiovascular disease, cancer, or diabetes requiring daily medication) and physical disabilities (e.g., musculoskeletal impairments or conditions that limit mobility and prevent participation in regular physical activity; *n* = 12), as these conditions could substantially affect both physical activity levels and depressive symptoms, potentially confounding the associations under investigation (Lubans et al., 2016); those without complete data on sedentary behavior, physical activity, depression, and covariates (*n* = 44); and those who did not wear their accelerometers for four valid days (including two weekdays and one weekend day; *n* = 157). Finally, a total of 521 participants were included in the study (70.98% response rate).

Considering sample representativeness and constraints on human and material resources, this study selected two public and material resources, this study selected two public junior high schools and one public senior high school in urban districts of Wuhan and Changsha, two megacities in China, for investigation. Both cities have permanent populations exceeding 10 million, high levels of urbanization. The participating schools are all large-scale public institutions (with over 1,000 students), featuring diverse socioeconomic backgrounds among students’ families, making them representative of typical urban adolescents. Overall, the selected cities and schools provide a reasonably representative sample of PA levels and related supportive environments among adolescents, establishing a solid foundation for the research.

### 2.2 Measures

#### 2.2.1 Physical activity and sedentary behavior

Those who participated in the PA and SED tests were objectively measured using Actigraph Model GT1M accelerometers (Pensacola, FL, United States). Participants were asked to wear the accelerometers on their right hip for seven consecutive days during all waking hours and outside of water-based activities. Accelerometer data were collected in 60-s epochs (intervals; Altenburg et al., 2021). ActiLife software, version 6.13.3, was used to filter and analyze the data. Non-wear time, defined as more than 20 min of consecutive zero counts, was excluded (Cliff et al., 2014). Additionally, participants were required to wear their accelerometers for at least 10 h per day (Colley et al., 2010), and for a minimum of four valid days within 1 week to be considered valid data (Cain et al., 2013). Evenson et al. (2008) cut points showed excellent classification accuracy in assessing PA and SED among adolescents (Trost et al., 2011). Therefore, we used this cut-points to categorize SED, LPA, and MVPA (Evenson et al., 2008). The average time spent on each activity was calculated by dividing the total time by the number of valid days. Accelerometer data were processed to exclude sleep periods, which were identified using validated algorithms combined with participants’ activity logs. Thus, PA and SED estimates reflect only wake time behaviors.

#### 2.2.2 Activity patterns

According to standardized definitions, we identified the activity patterns as the frequency and duration of PA and SED bouts accumulated during the waking day (Verswijveren et al., 2018; Ridgers et al., 2023). A PA bout was considered a continuous episode within a specific intensity range (Diaz et al., 2020). A sedentary bout is defined as a sustained and uninterrupted period of SED (Altenburg et al., 2015; Tremblay et al., 2017). Any bouts were continuous without interruptions, and the end of a bout was signified by an interruption in intensity (Verswijveren et al., 2020; Bueno et al., 2022). Therefore, based on previous literature (Saunders et al., 2013; Lätt et al., 2018; Santos et al., 2019; Verswijveren et al., 2020) and the exploration of participants’ activity patterns in this sample, the variables used to characterize the activity patterns of PA and SED include duration (min) and frequency (number) of 1–4 min SED, 5–9 min SED, 10–14 min SED, 15–29 min SED, ≥30 min SED, 1–4 min MVPA, 5–9 min MVPA, ≥10 min MVPA, 1–9 min LPA, 10–19 min LPA, ≥20 min LPA. In total, 22 variables were included, and the average daily duration and frequency for different bouts of SED, MVPA, and LPA were calculated. These variables were selected to provide a comprehensive representation of adolescents’ daily activity patterns. In contrast, previous studies predominantly analyzed PA or SED in isolation, overlooking their trade-off relationship in time allocation (Jakicic et al., 2019; Werneck et al., 2019). By simultaneously considering PA and SED bouts across varying durations and frequencies, this study offers a more nuanced characterization of activity accumulation, which may be more relevant to understanding health outcomes.

#### 2.2.3 Depressive symptoms

The Center for Epidemiologic Studies Depression Scale (CES-D) was employed to assess depressive symptoms in participants (Radloff, 1977). The CES-D has demonstrated strong psychometric properties in measuring adolescent psychological states (Radloff, 1991). Participants reported their depressive feelings over the past week through a questionnaire composed of 20 items across four dimensions: depressed affect, positive affect, somatic activity or retardation, and interpersonal relationships. A four-point scale was used, ranging from 0 (never or rarely) to 3 (most of the time), except those questions 4, 8, 12, and 16 are reverse-scored, while the remaining items are scored positively. The CES-D score ranges from 0 to 60, with higher scores indicating a greater likelihood of depression. The Cronbach’s *α* coefficient of this study was 0.87.

### 2.3 Confounders

Age, gender, parental educational level, family economic conditions, school grade, and average accelerometer wear time during waking hours were assessed based on participants’ self-reported questionnaires and included as covariates due to their demonstrated association with adolescents’ activity patterns and mental health (Huang and Wong, 2016; Cao et al., 2020). Parental educational level (middle school or less, high school, college/university above) and household income per person annually based on Chinese currency in RMB (<9,000, 9,000–30,000, 30,001-100,000, >100,000), were collected by participants asking their parents. School grade refers to junior high school and senior high school.

### 2.4 Statistical analyses

Statistical analyses were conducted using IBM SPSS software version 27.0 and Mplus version 8.3.

In the first step, 22 activity pattern variables were utilized in the latent profile models. Then, we fit successive latent profile models ranging from one to five profiles. The final number of profiles for the activity patterns was determined based on the following criteria: (1) entropy values greater than 0.8 indicated that the accuracy of individual classifications is greater than 90%, and the closer it is to 1, the more accurate the classification (Nylund et al., 2007; Jiang et al., 2024). (2) smaller values of the Akaike information criterion (AIC), Bayesian information criterion (BIC), and sample-size adjusted BIC (SABIC) indicated a superior fit of the model (Lo et al., 2001); (3) a significant *p*-value for both the Bootstrap likelihood ratio test (BLRT) and Lo–Mendell–Rubin (LMR) test indicated that the model provided a better fit than the previous model (Lubke and Neale, 2006); (4) each individual was assigned to distinct group models, with the smallest group comprising more than 5% of the total sample (Tein et al., 2013). Finally, to explore associations between groups of adolescent activity patterns and depressive symptoms were examined using linear regression analysis.

## 3 Results

### 3.1 Participants’ characteristics

A total of 521 adolescents were enrolled in this study. Of these, the mean age of the analytic sample was 14.73 years (SD = 1.30), and 53.9% of participants were female. Descriptions of parental education, household income, BMI, total daily volume, total daily frequencies, and depressive levels are presented in Table 1. Included (*n* = 521) and excluded (*n* = 213) adolescents were similar in terms of participants’ characteristics.

**Table 1:** Participants’ characteristics for the total sample and three activity patterns profiles.

Variables	Total sample (*n* = 521)	Prolonged sitters (*n* = 149)	Sitters (*n* = 224)	Movers (*n* = 148)
	%(*n*) or mean ± SD			
Age (years)	14.73 ± 1.30	14.76 ± 1.32	14.79 ± 1.32	14.61 ± 1.26
Sex (%)				
Girls	53.9 (281)	63.8 (95)	57.1 (128)	39.2 (58)
Boys	46.1 (240)	36.2 (54)	42.9 (96)	60.8 (90)
School grade				
Junior high school	53.2 (277)	55.7 (83)	50 (112)	55.4 (82)
Senior high school	46.8 (244)	44.3 (66)	50 (112)	44.6 (66)
Parental education (%)				
Middle school or less	49.1 (256)	53.7 (80)	46.0 (103)	49.3 (73)
High school	34.4 (179)	32.2 (48)	33.9 (76)	37.2 (55)
College/university above	16.5 (86)	14.1 (21)	20.1 (45)	13.5 (20)
Household income/person (RMB/year)				
<9,000	19.0 (99)	19.5 (29)	16.1 (36)	23.0 (34)
9,000–30,000	36.3 (189)	38.9 (58)	37.1 (83)	32.4 (48)
30,001-100,000	35.1 (183)	32.2 (48)	35.3 (79)	37.8 (56)
>100,000	9.6 (50)	9.4 (14)	11.6 (26)	6.8 (10)
Total daily volumes (min/day)				
SED	569.26 ± 111.68	573.01 ± 106.76	579.68 ± 113.65	549.72 ± 111.74
MVPA	29.75 ± 15.28	24.72 ± 11.65	29.55 ± 15.39	35.12 ± 16.60
LPA	173.67 ± 49.38	120.16 ± 23.71	171.99 ± 24.35	230.08 ± 32.39
Wear time	772.38 ± 113.67	720.21 ± 111.63	780.62 ± 109.14	812.44 ± 103.05
Total daily frequencies (number/day)				
SED	69.66 ± 15.76	51.12 ± 6.91	69.29 ± 6.49	88.89 ± 6.79
MVPA	14.08 ± 5.85	11.94 ± 5.14	14.23 ± 5.63	16.02 ± 6.15
LPA	79.69 ± 17.84	58.61 ± 8.74	79.39 ± 7.20	101.27 ± 7.39
Depressive symptom (score*)	15.89 ± 9.68	18.40 ± 9.28	15.71 ± 9.10	13.64 ± 10.37

SD, standard deviation; SED, sedentary behavior; MVPA, moderate to vigorous physical activity; LPA, light physical activity. *Higher scores represent sever degree of depression.

### 3.2 Determination of the number of latent profiles

The model fit indices for one to five class latent profile models are provided in Table 2. The results revealed that the three-class activity accumulation pattern yield the optimal model fit. The AIC, BIC, and SABIC values decreased with the addition of latent classes across the models with different numbers of latent classes, however, the rate of decrease became less pronounced after the number of three-class solution. The three-class solution demonstrated the highest classification entropy, with statistically significant LMR and BLRT values (*p* < 0.05). Thus, based on the interpretability of the classes, fit indices, and the sample size of the classes, the three-class model was selected as the most appropriate fit.

**Table 2:** Fit indices of latent profile models with one-to-five-class solutions.

Number of latent classes	AIC	BIC	SABIC	Entropy	*p*-value		Proportion in each class
					LMR	BLRT	
1	71846.167	72033.42	71893.755				
2	68908.983	69194.119	68981.446	0.942	<0.001	<0.001	283/238
**3**	**67683.001**	**68066.018**	**67780.338**	**0.955**	<**0.001**	<**0.001**	**149/224/148**
4	66807.728	67288.620	66929.941	0.952	0.3681	<0.001	139/76/205/101
5	66252.534	66831.316	66399.622	0.954	0.3061	<0.001	40/189/66/92/34

AIC, Akaike information criterion; BIC, Bayesian information criterion; SABIC, sample-size adjusted BIC; LMR, Lo–Mendell–Rubin; BLRT, Bootstrap likelihood ratio test. The optimal model was shown in bold.

### 3.3 Latent profiles descriptions

**Figure 1:**
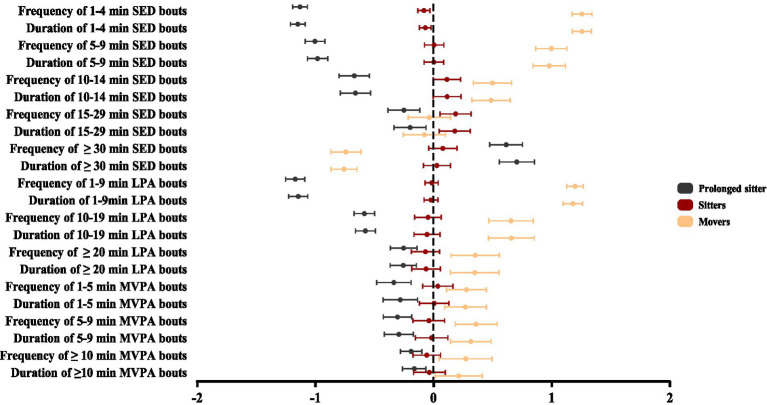
Standardized means (Z-scores with 95% confidence intervals) of activity pattern variables across three distinct groups.

The naming of classification results followed previous literature, identifying participant groups with similar bouts of SED and PA based on their most salient features relative to other patterns (Chen et al., 2024; Verswijveren et al., 2020). Participants with similar cumulative patterns were categorized into three distinct groups, with their high and low z values illustrated in Figure 1. The three distinct latent profiles were identified: “Prolonged sitters” (*n* = 149; 28.6%) demonstrated the greatest duration and highest frequency in ≥ 30 min SED bouts and the shortest durations and lowest frequencies in PA bouts. “Sitters” (*n* = 224; 43.0%) accumulated the longest duration and highest frequency in 15–29 min SED bouts, moderate PA bouts. “Movers” (*n* = 148; 28.4%) exhibited the longest durations and highest frequencies in PA bouts and <15 min SED bouts engagement. Prolonged sitters were considered as unhealthiest group and was used as a reference for comparisons, due to their longest duration, highest frequency in ≥30 min SED bouts, and lowest number of PA bouts.

### 3.4 Associations between distinct groups and depressive symptoms

Table 3 presents the associations between the three distinct groups and depressive symptoms. In adjusted models controlling for potential confounders (Model 2), compared with the prolonged sitters, the sitters [*β* (95% CI) = −2.3 (−4.240, −0.325), *p* = 0.022] and movers [*β* (95% CI) = −3.6 (−5.912, −1.388), *p* = 0.002] were a significant associated with a lower total depressive symptom, with an average reduction of 2.3 and 3.6 scores, respectively.

**Table 3:** Linear regression models of the associations between three distinct groups and depressive symptoms.

Activity patterns groups	Prolonged sitters*		Sitters		Movers	
	β (95% *CI*)	*p*	β (95% *CI*)	*p*	β (95% *CI*)	*p*
Model 1	Referent	-	**−2.7(−4.677, −0.718)**	**0.008**	**−4.8 (−6.941, −2.594)**	**<0.001**
Model 2	Referent	-	**−2.3(−4.240, −0.325)**	**0.022**	**−3.6 (−5.912, −1.388)**	**0.002**
Model 3	Referent	-	**−2.4 (−4.329, −0.385)**	**0.019**	**−3.8 (−6.091, −1.504)**	**0.001**
Model 4	Referent	-	−1.6 (−4.629, 1.477)	0.311	−2.1 (−7.636, 3.345)	0.443
Model 5	Referent	-	−1.6 (−4.531, 1.372)	0.294	−2.2 (−7.347, 3.017)	0.412
Model 6	Referent	-	−1.6(−4.690, 1.480)	0.307	−2.1(−7.693, 3.431)	0.452

*Represent referent group; β, regression coefficients; CI, confidence interval. Unadjusted model 1 included only activity patterns groups; Model 2 was adjusted for sex, age, parental educational level, family economic conditions, school grade, and average accelerometer wear time during waking hours; Model 3 was further adjusted total MVPA duration and frequency based on Model 2; Model 4 was additionally adjusted total SED duration and frequency based on Model 2. Model 5 was additionally adjusted total LPA duration and frequency based on Model 2. Model 6 was fully adjusted total MVPA duration and frequency, total LPA duration and frequency, and total SED duration and frequency based on Model 2. Significant associations are showed in bold.

When Model 3 was further adjusted for MVAP duration and frequency based on Model 2, the reduction in depressive symptoms observed in the sitters [*β* (95% CI) = −2.4 (−4.329, −0.385), *p* = 0.019] and movers [β (95% CI) = −3.8 (−6.091, −1.504), *p* = 0.001] remained similar those in Model 2, compared with the prolonged sitters.

However, when Model 4 was additionally adjusted for total SED duration and frequency based on Model 2, neither the sitters [β (95% CI) = −1.6 (−4.629, 1.477), *p* = 0.311] nor the movers [β (95% CI) = −2.1 (−7.636, 3.345), *p* = 0.443] showed statistically significant differences in depressive symptoms compared to the Prolonged sitters. Additionally, after further adjusted total LPA duration and frequency in Model 5 (based on Model 2), we found no statistically significant differences in depressive symptoms between either the sitters [β (95% CI) = −1.6 (−4.531, 1.372), *p* = 0.294] or the movers [β (95% CI) = −2.2 (−7.347, 3.017), *p* = 0.412] and the prolonged sitters group. Similarly, after simultaneously controlling for total SED duration and frequency, total LPA duration and frequency, and total MVPA duration and frequency based on Model 2, no statistically significant differences were found between the sitters group [β (95% CI) = −1.6 (−4.690, 1.480), *p* = 0.307] or the movers group [β (95% CI) = −2.1 (−7.693, 3.431), *p* = 0.452] showed no statistically significant differences compared to the prolonged sitters group.

## 4 Discussion

This study presents the first application of latent profile approach to examine the association between activity patterns, characterized by duration and frequency of PA and SED, and depressive symptoms among adolescents aged 13–18 years. Three distinct profiles of activity patterns were identified and labeled as Prolonged sitters, Sitters, and Movers. After adjusting for potential confounders, Movers, characterized by the longest duration and highest frequency of PA bouts and <15 min SED bouts, demonstrated the lowest total depressive symptoms compared with Prolonged sitters, followed by Sitters accumulating the longest duration and highest frequency of 15–29 min SED bouts. Together, these findings highlight the differences in activity accumulation patterns among adolescents and suggest that targeted interventions should address specific behavioral groups to promote better mental health outcomes.

Analysis of the proportion of individuals across different activity pattern groups reveals that the “movers” group had the lowest proportion at only 12.0%, followed by the “prolonged sitter” at 28.6%. The “sitters” group had the highest proportion 43%. This indicated that the highest 15–29 min SED bouts represent the most typical activity accumulation patterns among the participating adolescents. This may reflect the school-based context of adolescents’ daily routines, where prolonged sitting during classes is common and opportunities for sustained PA are limited (Contardo Ayala et al., 2024; Penning et al., 2017).

After controlling for potential confounders, adolescents in sitters and movers exhibited lower depressive symptoms levels compared to Prolonged Sitters, and the protective effect was more pronounced in movers. On the one hand, sitters and movers experienced more frequent interruptions of SED and bouts of SED typically lasted less than 30 min. Extensive evidence from prior studies has established a robust association between prolonged SED (i.e., uninterrupted SED bouts ≥30 min) and negatively cardiometabolic outcomes (Cliff et al., 2014; Lätt et al., 2018; Rodriguez-Ayllon et al., 2019; Carlson et al., 2023), as well as elevated systemic inflammation levels (Sha et al., 2024; Chen et al., 2025). These adverse physiological factors may be involved in the development of depressive symptoms by their detrimental effects on neurovascular function and neurotransmitter systems. On the other hand, the movers’ group had higher frequency and duration of bouts of MVPA/LPA compared to the sitters, especially in LPA. These frequent short durations of MVPA or LPA may further improve cardiovascular health by interrupting SED (Chastin et al., 2015; White et al., 2015; Fernstrom et al., 2023). These mechanisms help mitigate the negative metabolic and inflammatory effects of prolonged sitting and may provide a protective effect against depressive symptoms through this pathway (Belcher et al., 2015; Chastin et al., 2015; Bates et al., 2021; Sha et al., 2024). However, it is important to note that the relationship between PA and depressive symptoms may be bi-directional. While lower levels of PA have been associated with higher depressive symptoms, adolescents experiencing depressive symptoms may also be less likely to participate in PA due to reduced motivation, fatigue, and social withdrawal (Biddle et al., 2019; Schuch et al., 2018). Our study further supports this mechanism, suggesting that sitters and movers’ patterns may help to reduce the risk of depression in adolescents. Therefore, the present study supports the idea that limiting less than 30 min and interrupting it with PA of any intensity and duration can help to improve the mental health of adolescents.

Furthermore, after controlling for potential confounders and total daily MVPA volume and frequency, Sitters and Movers exhibited favorable differences in depressive symptom outcomes compared to Prolonged sitters. The reason that total MVPA and frequency did not fully explain the differences in depression may have been the low total MVPA participation of adolescents in this study, which prevented it from counteracting the negative effects of prolonged SED on depression (Healy et al., 2008; Jiang et al., 2023; Hou et al., 2025). However, the Sitters and Movers group, characterized by frequent interruptions of sedentariness and shorter bouts of PA, is consistent with the findings of existing studies that their pattern of activity accumulation is an important factor in improving health (Verswijveren et al., 2018; Verswijveren et al., 2020).

However, after adjusting for confounders and total SED and total LPA, the associations between activity patterns and depressive symptoms were no longer significant. This finding suggests that for Chinese adolescents, reducing total daily SED volume and ensuring sufficient LPA may be more fundamental and critical for preventing depression than merely altering the activity accumulation patterns (e.g., increasing break frequency; Duvivier et al., 2013). With the unique educational context in China, heavy academic demands, and prolonged continuous sedentary study being extremely common (Zhu et al., 2019). This “compulsory sitting” may be accompanied by greater psychological stress. Therefore, any intervention that reduces total SED time, even if achieved by increasing LPA, may yield mental health benefits by alleviating perceived stress and enhancing physical arousal.

A key finding of this study is that when controlling for both the total PA and SED simultaneously, the association between activity patterns and depressive symptoms no longer reached statistical significance. This suggests that the underlying mechanisms driving health risk differences across distinct activity patterns may be primarily mediated by the aggregate levels of SED, LPA, and MVPA, rather than the temporal distribution patterns themselves. This result carries important public health implications. It indicates that preventive interventions for adolescent depressive symptoms should prioritize reducing overall SED and increasing total PA levels. Such strategies may be more universal and cost-effective than focusing solely on complex “pattern” modifications (e.g., strict requirements to interrupt SED). Nevertheless, this does not mean that patterns are irrelevant. The associations remained significant after adjusting for MVPA and confounders, suggesting that the “Movers” group may benefit from protective factors beyond MVPA volume, such as higher activity intensity, greater social interaction, or the positive psychological experiences associated with exercise areas worthy of further investigation (Schuch et al., 2018).

A significant strength of person-centered analyses is the potential to inform tailored intervention strategies for identified subgroups. By identifying distinct activity accumulation patterns, our results further illustrate this advantage and indicate that interventions should be implemented according to group characteristics. For “prolonged sitters,” interventions should prioritize interrupting prolonged sitting (e.g., ≥30 min SED) through short PA bouts. For “sitters,” strategies may focus on reducing 15–29 min SED bout and gradually increasing PA engagement. For “movers,” the emphasis should be on maintaining and reinforcing their already favorable activity patterns. Such a tailored approach may enhance the effectiveness and sustainability of adolescent mental health promotion programs.

This study also has several limitations. Firstly, this study was cross-sectional, we were unable to establish the direction of association between activity patterns and depressive symptoms. Future longitudinal and intervention studies are needed to clarify these potential bi-directional pathways. Secondly, while the present study used objective accelerometers to measure adolescents’ activity patterns, it did not include any self-reported measures to indicate the types of activities performed or the contexts in which they occurred. The absence of self-reported data also precluded triangulation with the accelerometer findings, which would have enhanced confidence in the results. Future research should combine self-report and objective measures to explore adolescents and their association with depressive symptoms (Ridgers et al., 2012). Thirdly, the test subjects in this study were all from China, the representativeness of the sample is limited, the sample was drawn from only three schools in Wuhan and Changsha, two urban areas in central China. Given the limited geographic scope, the findings cannot be considered nationally representative of all Chinese adolescents. Future studies should include multiple regions and rural areas to improve the generalizability of the results. Lastly, body mass index (BMI) and certain other individual-level factors were not included as covariates in the analyses. As BMI has been shown to be associated with both physical activity patterns and mental health outcomes, future studies should consider including BMI and other relevant covariates to better control for potential confounding effects.

## 5 Conclusion

In summary, based on latent profile analysis, this study identified three distinct activity pattern groups: prolonged sitters, sitters, and movers. Additionally, adolescent depression risk disparities across activity pattern groups may be largely attributable to differential exposure to SED and LPA (both duration and frequency). Therefore, fragmenting prolonged sedentariness and habitual LPA integration may be promising prevention targets, pending verification in longitudinal or interventional research.

## Data Availability

The raw data supporting the conclusions of this article will be made available by the authors, without undue reservation.
